# Intimate Partner Violence and HIV Sexual Risk Behaviour Among Women Who Inject Drugs in Indonesia: A Respondent-Driven Sampling Study

**DOI:** 10.1007/s10461-018-2186-2

**Published:** 2018-06-11

**Authors:** Claudia Stoicescu, Lucie D. Cluver, Thees Spreckelsen, Marisa Casale, Anindita Gabriella Sudewo, Irwanto

**Affiliations:** 1 Centre for Evidence-Based Intervention, Department of Social Policy and Intervention, University of Oxford, Barnett House, 32 Wellington Square, Oxford, UK; 2 Department of Psychiatry and Mental Health, University of Cape Town, Cape Town, South Africa; 3 School of Public Health, University of the Western Cape, Cape Town, South Africa; 4 HIV and AIDS Research Centre, Atma Jaya Catholic University, Jakarta, Indonesia; 5 Kirby Institute, University of New South Wales, Sydney, Australia

**Keywords:** Intimate partner violence, HIV, Sexual risk behavior, Women, Injecting drug use, Respondent-driven sampling

## Abstract

Women who inject drugs are disproportionately affected by HIV and intimate partner violence (IPV); however, the link between IPV and HIV remains under-researched among substance-using women in low- and middle-income countries. This study examined associations and additive effects of different forms of IPV victimization (psychological, physical and/or injurious, and sexual) on HIV sexual risk behavior among women who inject drugs in Indonesia. Respondent-driven sampling (RDS) was used to recruit 731 women from Greater Jakarta and Bandung, West Java. RDS-II weighted prevalence of any past-year IPV was 68.9% (95% CI 65.0, 72.6) in Jakarta and 55.9% (95% CI 48.0, 63.5) in Bandung. In separate logistic regressions controlling for socio-demographic covariates, all three forms of IPV showed statistically significant associations with sexual risk behavior. After adjusting for all IPV types, psychological (OR 1.87; 95% CI 1.17, 2.99; p = 0.009) and sexual (OR 1.98; 95% CI 1.22, 3.21; p = 0.006) IPV independently predicted women’s sexual risk behavior. Marginal effects models suggested that co-occurrence of multiple forms of IPV had greater adverse consequences: sexual risk behavior was reported by 64.1% of women who did not experience any IPV, but increased to 89.9% among women exposed to all three types. Comprehensive harm reduction services that integrate IPV monitoring and prevention are urgently needed to reduce both HIV and IPV.

**Electronic supplementary material:**

The online version of this article (10.1007/s10461-018-2186-2) contains supplementary material, which is available to authorized users.

## Resumen

Las mujeres que se inyectan drogas se ven desproporcionadamente afectadas por el VIH y la violencia de pareja (IPV); sin embargo, el vínculo entre la IPV y el VIH sigue siendo poco investigado entre las mujeres que usan sustancias en países de bajos y medianos ingresos. Este estudio examinó las asociaciones y los efectos aditivos de las diferentes formas de victimización por IPV (psicológica, física y/o perjudicial y sexual) en el comportamiento de riesgo sexual del VIH entre las mujeres que se inyectan drogas en Indonesia. Se utilizó el muestreo dirigido por el encuestado (RDS) para reclutar a 731 mujeres del Gran Yakarta y Bandung, Java Occidental. La prevalencia ponderada de RDS-II de cualquier IPV de años anteriores fue de 68.9% (IC 95% 65.0, 72.6) en Yakarta y 55.9% (IC 95% 48.0, 63.5) en Bandung. En regresiones logísticas separadas que controlan las covariables sociodemográficas, las tres formas de IPV mostraron asociaciones estadísticamente significativas con el comportamiento de riesgo sexual. Después de ajustar para todos los tipos de IPV, psicológico (OR 1.87, IC 95% 1.17, 2.99, p = 0.009) y sexual (OR 1.98, IC 95% 1.22, 3.21, p = 0.006) IPV predijo de forma independiente el riesgo sexual de las mujeres comportamiento. Los modelos de efectos marginales sugirieron que la concurrencia de múltiples formas de IPV tuvo mayores consecuencias adversas: el 64.1% de las mujeres que no experimentaron ninguna IPV informaron el comportamiento de riesgo sexual, pero aumentó al 89.9% entre las mujeres expuestas a los tres tipos. Se necesitan con urgencia servicios integrales de reducción de daños que integren el monitoreo y prevención de IPV.

## Introduction

Asia is home to half of the estimated 3.8 million women who inject drugs globally [1]. Meta-analytic evidence has established that women who inject drugs experience higher levels of HIV than their male counterparts in high prevalence settings [2]. At the same time, there is growing recognition that social and structural factors shape individual risks and increase vulnerability to HIV via direct and indirect pathways [3, 4]. Intimate partner violence (IPV) has been highlighted as a key contributor to HIV transmission risk among drug-using women [5]. IPV is also more prevalent among women who inject drugs vis-à-vis women in the general population. For example, a recent review identified rates of past-year IPV ranging from 20% to 57% among clinical and community-based samples of women who use drugs in the United States, which is 2–5 times higher than prevalence rates found among general female populations [6, 7].

In North America, IPV victimization against women who inject drugs has been associated with the presence of multiple risk factors for sexually-transmitted HIV, including condomless sex, multiple sexual partners, history of past or current sexually transmitted infections (STIs), and trading sex for money, drugs, or shelter [8]. Different forms of IPV (i.e. psychological, physical, and sexual) may increase women’s susceptibility to HIV risk through direct and indirect mechanisms. Sexual IPV or forced sex may directly exacerbate women’s HIV risk through biological mechanisms, via genital injuries and lacerations that facilitate disease transmission [9]. Indirectly, both sexual and physical IPV have been shown to impact HIV risk by creating a dynamic of fear and submission that hinders a woman’s ability to negotiate safer sex [5]. Psychological abuse may create a similar context of dominance and control, which increases women’s likelihood of engaging in risky sexual behaviors [10]. In fact, emerging research suggests that psychological aggression has similar detrimental effects on women’s health outcomes to physical and sexual forms of IPV [11, 12].

A growing body of international research has documented strong associations between IPV and HIV, both in the general population [13] and among key populations such as men who have sex with men and female sex workers [14]. However, extant research investigating this association among drug-involved women is geographically clustered in high-income countries [5, 15, 16]. Crucially, no published research to date has explored this relationship among women who inject drugs in low- and middle-income countries in Asia.

In contrast with a trend of stabilisation across most countries in the Asia region, Indonesia is facing an escalating HIV epidemic concentrated among key populations [17]. With an HIV prevalence of 36.4%, people who inject drugs remain disproportionally affected compared with other key populations, such as female and transgender sex workers and their clients, and men who have sex with men [18]. Despite their smaller numbers compared with their male counterparts, women who inject drugs face elevated vulnerability to HIV [19–21]. In 2009, the only year for which sex-disaggregated estimates are available, HIV prevalence among women who inject drugs in Indonesia was 57.1%, relative to 52.1% among male injectors [22]. Furthermore, qualitative studies from urban settings across the Indonesian archipelago suggest that IPV and HIV vulnerability may co-occur among women who use and inject drugs [19, 20, 23–25]. For instance, in a multi-city qualitative study of 52 women who use drugs, Habsari et al. identified pervasive exposure to violence perpetrated by both intimate and non-intimate partners [19]. Women have also reported that in situations where they felt at risk of HIV infection whilst in an abusive relationship, their priority was not protection against HIV infection. Instead, women sought to avoid conflict out of fear of provoking aggression from their partners, and a desire to “maintain their relationship” [25]. However, no quantitative studies to date have explored the prevalence and associations of IPV and HIV sexual risk behavior among women who inject drugs in Indonesia.

Understanding the relationship between different forms of IPV and sexual risk behavior is essential for elucidating pathways to HIV and for informing effective interventions with women who inject drugs in low-and middle-income countries. There is a clear need for quantitative research with adequately sized samples and validated measures, to assess the effect of IPV on drug-using women’s HIV risk outcomes in Asia. Accordingly, this study examines the largest known sample of Indonesian women who inject drugs to date to investigate (1) associations between exposure to psychological, physical and/or injurious, and sexual dimensions of IPV and HIV sexual risk behavior; and (2) potential additive effects of IPV polyvictimization on women’s sexual risk behavior.

## Methods

### Study Design and Sampling

A cross-sectional design was employed to recruit women who inject drugs from urban areas with large numbers of people who inject drugs and high HIV rates among injectors [26]: Jakarta and peri-urban surrounding cities Bogor, Tangerang, Depok and Bekasi (hereafter referred to as “Greater Jakarta”) and Bandung, the provincial capital of West Java. The minimum sample size for this study (709 participants) was calculated by assuming a 36% HIV prevalence rate [18], with 95% confidence, 5% precision, and a design effect of 2 [27, 28].

The community of women who inject drugs was actively engaged in the development, implementation, and dissemination of the research. Four focus group discussions with a total of 39 women, and three consultations with relevant key population networks and community-based organisations, were convened to ensure that all study procedures were sensitive to the needs of participants. A community advisory group comprised of six women with an injecting drug use background was established to advise researchers throughout study implementation. Once the data were analysed, a consultation was convened to discuss results with peers and devise strategies for dissemination.

Between September 2014 and June 2015, 731 women were recruited using respondent-driven sampling (RDS). RDS, a modified chain referral sampling method, is known to be effective for the recruitment of populations that are hard-to-reach [29]. Akin to snowball sampling, RDS utilizes peer networks to recruit participants. However, RDS limits the influence of recruiters on the final composition of the sample by restricting the number of recruits per recruiter, and weights the sample by participants’ probability of recruitment (social network size) to adjust for non-random sampling [30]. Since its introduction in 1997, over 460 RDS studies in 69 countries have been conducted with hidden or hard-to-reach populations [31].

Eligibility criteria included: being ≥ 18 years of age; residing in one of the study catchment areas; injecting drugs in the preceding 12 months; and possessing a valid recruitment referral. Guided by the internationally-supported definition proposed by WHO, UNODC, and UNAIDS and by indicators used in national surveillance [26, 32], this study deemed women reporting any instance of illicit or illegal drug injecting occurring in the previous 12 months as eligible [33].

### Procedures

To initiate recruitment, a diverse group of 20 initial recruits (“seeds”) was selected by the researchers. Seed selection was informed by extensive formative research, including mapping of hotspots where people inject drugs, key informant interviews with local harm reduction service providers, and community consultations. To increase the representativeness of the sample, selected seeds were heterogeneous in terms of age, education, levels of risk behavior, and known HIV status. Each initial recruit was asked to refer up to three peers to the study, who in turn enlisted others in a chain-referral fashion. Successive waves of recruitment continued until the desired sample size was reached.

Questionnaires were translated into Bahasa Indonesia by bilingual health workers and pre-tested with women representative of the target sample according to WHO guidelines [34]. Seven female peer fieldworkers were trained by senior researchers in mobile-assisted interviewing, ethics, and health and safety. Face-to-face interviews lasted approximately 1 h and were conducted in the local language at locations deemed safe by participants, such as offices of non-governmental organisations or participants’ homes. Information was collected using tablets equipped with Open Data Kit, an open-source application for data collection and management on mobile devices [35].

The study used mobile-site interviewing. Potential recruits were asked to contact the research team by phone or text message to set up an interview at a location of their choice. As part of the RDS process, participants received a primary incentive of 75,000 Indonesian Rupiah (~ USD $5) for participating in the interview and a secondary incentive of 25,000 Indonesian Rupiah (~ USD $2) per eligible peer recruited. Monetary remuneration is considered an ethical and effective way to facilitate participation in public health research by people who use drugs [36, 37]. Appropriate renumeration was determined by consulting the community advisory group and previous bio-behavioral surveys with people who inject drugs in Indonesia. Each recruit was given a uniquely coded identifier and recorded in SyrEx2, a monitoring and evaluation tool used by drug service providers [38].

### Ethical Considerations

The study was anonymous, and all participants were encouraged to use a pseudonym. Verbal and written voluntary informed consent was obtained from each participant. Consent forms were worded in plain language and included clear explanations of the nature and purpose of the research, limits to confidentiality in the context of illegal activities, and explicit statements regarding participants’ rights to opt-out at any point. Consent forms were read and discussed verbally by the interviewers to ensure that participants had the necessary information to be able to provide informed consent, regardless of literacy level.

Strict confidentiality was maintained, except where participants requested assistance or service referrals. In the case that information disclosed suggested that a participant was at risk of significant harm (e.g. severe violence) the interviewer discussed concerns with the participant and offered service referrals. Researchers maximised opportunities for referral by providing all participants with a local directory of HIV/STI testing and counselling, legal aid, and IPV support services. Interviewers explained to participants what existing services they could access for free and how to do so. Ethical protocols were approved by the ethics boards at the University of Oxford (ref no: SSD/CUREC2/13-23) and Atma Jaya University (ref no: 1114/III/LPPM-PM.10.05/11/2013).

### Measurement

#### HIV Sexual Risk Behavior

HIV sexual risk behavior in the preceding 12 months was measured using items from the UNAIDS Global AIDS Progress Reporting Indicator Registry [39] and informed by WHO guidance [40]: (1) condomless sex at last vaginal and/or anal intercourse; (2) multiple sexual partners; and (3) STI symptomatology. Sexually active participants were asked the following yes/no question, “Think about the last time you had vaginal and/or anal sex with any sexual partner. Did you use a condom the last time you had sex?” Participants who responded in the negative were coded as having had condomless sex at last intercourse (0 = used condom/not sexually active; 1 = did not use condom at last intercourse). Participants were also asked about the total number of sexual partners in the preceding 12 months. Following previous research [8, 41], *multiple sexual partners* was operationalized as having two or more sexual partners in the previous year. *Condomless sex at last intercourse* and *multiple sexual partners* included both steady and casual partners and paid and unpaid sex. *STI symptomatology* was assessed using a multiple-choice checklist of six easily recognised symptoms (i.e. “burning sensation and/or discomfort when urinating,” “itching, irritation and/or discomfort in the genital area,” “discomfort and/or pain during sexual intercourse,” “sores, blisters and/or ulcers on or in the vagina,” “unusual vaginal discharge, such as pus or a thick and/or sticky liquid from the genital area,” and/or “lower abdominal pain”) [40, 42]. STI symptomatology was ascertained if participants reported experiencing ≥ 2 symptoms.

A dichotomous variable reflecting women’s *HIV* *sexual risk behavior* during the preceding 12 months was created by coding one or more affirmative responses to the three items above as the presence of sexual risk behavior (0 = no sexual risk behavior, 1 = sexual risk behavior). Participants who did not endorse any of the three risk behaviors assessed and those who were not sexually-active were coded as 0 = no sexual risk behavior.

#### Intimate Partner Violence

*Intimate partner violence* was assessed using the psychological, physical, injurious, and sexual subscales of the Revised Conflict Tactics Scale (CTS2) short form [43, 44]. The CTS2 has been cross-culturally validated in more than 17 countries, including several low- and middle-income countries in Asia [45], and is the most widely-used measure assessing IPV victimization in community and clinical samples of drug-using women [8, 41, 46]. Sample items from each of the subscales include: psychological aggression (“My partner insulted or swore or shouted or yelled at me”); physical assault (“My partner punched or kicked or beat-me-up”); injurious physical assault (“I went to see a doctor or needed to see a doctor because of a fight with my partner”); and sexual coercion (“My partner used force, like hitting, holding down, or using a weapon, to make me have sex”). Participants were asked about violence perpetrated by a current or former intimate partner in the preceding 12 months. Guided by previous research [47, 48], binary variables were created for each type of IPV (psychological, physical and/or injurious, and sexual) by assigning a score of *1* if one or more instances of the items were reported to have occurred in the past year and *0* if no instances were reported. Affirmative responses to IPV victimization items on each subscale were coded as *1* regardless of responses to subscales for other IPV types. For this sample the CTS2 subscales showed adequate to high internal consistency, ranging between α = 0.65 and α = 0.82, and totalling α = 0.87 for the full scale.

#### Sociodemographic and Background Characteristics

Informed by a literature review and formative research, selected socio-economic and background information was collected as the basis for a confounder analysis and potential effect modification [8, 20, 41, 49]. Using items modelled on the Indonesia Population Census (Statistics Indonesia) and Integrated Biological and Behavioral Surveillance (Ministry of Health), women were asked their *age, relationship status, employment status, level of education, individual monthly income, and whether they had any dependent children* in the household or other dependents for whom they were responsible. *Individual monthly income* was classified as being either below or above the mean national income in Indonesia [50]. Participants were also asked about illegal and/or illicit drug use in the previous 12 months, and whether they had knowledge of their HIV status.

Since previous longitudinal research has established a link between drug-using women’s financial dependency on their intimate partner and elevated sexual risk behavior [51], a variable reflecting this construct was included as a potential confounder. *Financial dependency* was assessed by asking participants about the main source (> 50%) of their monthly income. Women were coded as financially dependent if they indicated that their main source of income was from intimate partner(s). Furthermore, studies have shown that the syndemic co-occurrence and interaction of multiple psychosocial factors may augment HIV risk-taking behavior [5, 52, 53]. In particular, as crystal meth has been shown to co-occur with IPV and heighten HIV risk [54, 55], a dichotomous variable was computed to indicate any past-year use of non-injection crystal meth. Following previous research indicating that the relationship between IPV, other syndemic factors, and HIV risk may be modified by structural influences such as poverty and housing instability [56, 57], a variable reflecting women’s housing status was also included. *Housing status* was assessed by asking participants about their current living arrangements and dichotomised into “stable housing” vs “unstable housing/homelessness”. Women were coded as “unstably housed and/or homeless” if they lived on the street, including in public spaces (i.e. train station) or in temporary or transitional accommodation, such as a friend’s home, and “stably housed” if they lived in their family home, rental house/apartment, and rental long-term single-room accommodation (*kos*-*kosan*). All measures were based on self-report.

### Statistical Analysis

Analyses were conducted in four stages:

1. Frequencies for all variables were conducted on the unweighted, aggregated sample. RDS-II weighted estimates of population proportions and 95% confidence intervals (CIs) and sample diagnostics were calculated using the user-written RDS analysis package [58, 59] in Stata 14 (StataCorp, College Station, TX). Preliminary analyses revealed that participants formed two isolated geographical components with minimal across-group recruitment (i.e. bottleneck), which can add variance to a sample and produce unstable estimates [60] (see Supplementary Appendix II). In the presence of bottlenecks, standard practice in RDS literature is to produce estimates for each sub-sample individually rather than combining them into an overall sample [60]. Therefore, weighted estimates and RDS diagnostics for HIV sexual risk and IPV variables were computed separately for each study city. However, in order to retain the power and precision corresponding to the initial calculated sample size, and because city differences can be adjusted for, the unweighted city sub-samples were aggregated for subsequent bivariate and multivariate analyses.
2. Bivariate associations between IPV and background variables and the sexual risk behavior outcome were examined using logistic regressions. Variables associated with sexual risk behavior at *p *< 0.1 were retained in multivariate analyses [61].
3. To explore the independent effects of each IPV dimension from its overall effect, separate multivariate logistic regression models were created for each form of IPV (psychological, physical and/or injurious, and sexual), and a final model controlled for all IPV dimensions simultaneously. For robustness, we assessed potential multicollinearity between predictor variables using variance inflation factor (VIF) diagnostic tests, which quantify how much the variance of the estimated regression coefficient is inflated by the presence of correlation among the independent variables in the model [62, 63]. The presence of multicollinearity was defined as tolerance values below 0.1 and VIF values equal to or greater than 10 [62]. For models 1–3 assessing each IPV dimension separately, VIFs for all independent variables were lower than 4, mean VIFs for all models were under 2, and tolerance values ranged between 0.25 and 0.89. In model 4, which included all IPV dimensions, VIFs for predictor variables ranged between 1.14 and 4.10. The mean VIF for the entire model was 2.29, which is lower than the accepted threshold of 6, thus indicating acceptable fit of the model. In addition, tolerance values for variables in model 4 were between 0.24 and 0.88, with no values falling below 0.1. Therefore, no evidence of multicollinearity was identified between the predictors analyzed in the present study. We also tested for plausible two-way interactions between each dimension of IPV and background variables using product terms. No statistically significant interactions were detected.
4. We tested whether sexual risk behavior effects were greater if women experienced more than one form of IPV. All IPV variables that were included in model 4 were entered into a marginal effects model, adjusting for significant confounders. Predicted probabilities of engaging in HIV sexual risk behavior under each potential combination of IPV exposures were computed, with significant covariates held at mean values.

## Results

### RDS Sample Characteristics

A total of 731 women who inject drugs were recruited into the study, using 18 seeds and 554 recruits in Greater Jakarta (n = 572) and 2 seeds and 157 recruits in Bandung (n = 159). Five seeds, two in Bandung and three in Greater Jakarta, generated 54% (n = 391) of the combined sample across the two survey cities. The largest recruitment chain reached up to 11 waves and contained 105 participants in Greater Jakarta, and 8 waves with 98 participants in Bandung. Participants’ mean personal network size (*degree*) was 4.7 (SD = 4.2, range 1–35) in Greater Jakarta, and 3.9 (SD = 2.0, range 1–21) in Bandung. No considerable differentials in mean degree were observed for any of the variables in this study.

An RDS sample attains equilibrium when the sample distribution on key variables remains stable (i.e. within 2% of cumulative sample proportions) as new recruits are added [64]. Convergence refers to the required referral chain length (i.e. depth) necessary to reach equilibrium [65]. To determine whether recruitment chains converged to a sampling equilibrium, convergence plots superimposing the weighted sample proportions at each recruitment wave on the cumulative proportion based on the complete sample were examined for select variables (see Supplementary Appendix I) [66]. Proportions for this sample appear to stabilize between waves 3–5 for Bandung and waves 2–5 for Greater Jakarta and remain stable until the full sample size is attained, indicating that the sample is becoming random as additional participants enroll.

Homophily assesses the extent to which participants prefer to recruit those with similar characteristics to themselves rather than uniformly at random [64]. We used the homophily index (Hx) proposed by Heckathorn, which contains values ranging from − 1.0 to 1.0 [30]. Scores close to 0 indicate random recruitment and scores higher than 0.3 (or − 0.3) specify substantial in-group contact. Most analysis and outcome variables showed low homophily (all Hx < 0.27), indicating a high tendency to recruit others at random. Moderate homophily was detected for sexual coercion (Hx = 0.35), such that women from Jakarta who did not experience past-year sexual coercion tended to recruit others like themselves 35% of the time and at random 65% of the time.

### Sociodemographic and background characteristics

Mean age in the aggregated, unweighted sample was 31.3 years (SD = 5.10 years) (Table 2). 20.2% of women completed less than a high school education, 38.7% were currently married, and 56.5% had children or other dependents for whom they were responsible. Mean individual monthly income was IDR 4.3 million/USD 385 (SD = 3.38), with more than half (54.5%) of participants earning less than the average national income (IDR 3.8 million ~ USD 285). Nearly half of the women (44.3%) were unemployed, and at least one quarter (25.4%) were financially dependent on an intimate partner. 5.3% of the women were homeless or unstably housed.

Drugs injected in the previous year included heroin (94.4%), illicit buprenorphine (19.2%), illicit pharmaceuticals (i.e. largely in the opiate and benzodiazepine class of substances, used without a prescription) (4.0%), and crystal methamphetamine (crystal meth) (0.8%). Drugs used via non-injection routes of administration, such as smoking, snorting, or swallowing, included heroin (93.8%), crystal meth (67.2%) illicit pharmaceuticals (46.2%), cannabis (36.3%), and ketamine (6.6%). Self-reported HIV prevalence in the sample was 46.7%.

### Prevalence of Sexual Risk Behavior and Intimate Partner Violence

Overall, similar proportions of participants in Greater Jakarta (76.2%; 95% CI 71.4, 80.5) and Bandung (74.1%; 95% CI 66.2, 80.6) reported one or more of three HIV sexual risk behaviors (i.e. condomless sex at last intercourse, STI symptomatology, or multiple sexual partners) (Table 1). The prevalence of condomless vaginal and/or anal sex at last intercourse ranged from 46.9% (95% CI 39.2, 54.8) in Bandung to 65.1% (95% CI 61.1, 68.8) in Greater Jakarta. The prevalence of STI symptomatology was higher in Greater Jakarta (52.8%; 95% CI 48.6, 56.8) relative to Bandung (21.2%; 95% CI 15.7, 28.1). However, a higher proportion of participants in Bandung reported having multiple sexual partners in the preceding year (37.8%; 95% CI 30.5, 45.7), compared with participants from Greater Jakarta (25.1%; 95% CI 21.6, 28.9) (Table 2).

**Table 1:** RDS-weighted estimations and 95% confidence intervals (CIs) for IPV victimisation and sexual risk behaviors among women who inject drugs in the Perempuan Bersuara study, by survey city

	Greater Jakarta (n = 572)				Bandung (n = 159)			
	N	Unweighted %	RDS-weighted %	95% CI	N	Unweighted %	RDS-weighted %	95% CI
Past-year intimate partner violence (IPV)								
Any type of IPV (psychological, physical/injurious and/or sexual)								
Yes	401	70.1	68.9	65.0, 72.6	91	57.2	55.9	48.0, 63.5
No	171	29.9	31.1	27.3, 35.0	68	42.8	44.1	36.5, 52.0
Psychological aggression								
Yes	348	60.8	58.8	54.7, 62.8	87	54.7	52.6	44.7, 60.3
No	224	39.2	41.2	37.2, 45.3	72	45.3	47.4	39.7, 55.3
Physical and/or injurious assault								
Yes	250	43.7	38.8	35.0, 42.8	75	47.2	42.2	34.8, 50.0
No	322	56.3	61.2	57.2, 65.0	84	52.8	57.8	50.0, 65.2
Sexual coercion								
Yes	192	33.6	39.8	35.7, 44.0	25	15.7	19.6	13.7, 27.3
No	380	66.4	60.2	56.0, 64.3	134	84.3	80.4	72.7, 86.3
HIV sexual risk behaviors								
Condomless vaginal and/or anal sex at last intercourse								
Yes	353	61.7	65.1	61.1, 68.8	86	54.1	46.9	39.2, 54.8
No	219	38.3	34.9	31.2, 38.8	73	45.9	53.1	45.2, 60.8
Sexually transmitted infection (STI) symptomatology (≥ 2 symptoms)								
Yes	287	50.2	52.8	48.6, 56.8	37	23.3	21.2	15.7, 28.1
No	285	49.8	47.2	43.2, 51.3	122	76.7	78.8	71.9, 84.3
Number of sex partners (past 12 months)								
≤ 1	334	58.4	63.8	59.8, 67.6	94	59.1	59.4	51.5, 66.8
2–5	138	24.1	25.1	21.6, 28.9	60	37.7	37.8	30.5, 45.7
≥ 6	100	17.5	11.1	9.2, 13.4	5	3.1	2.8	1.1, 6.6
Endorsement of one or more of the sexual risk behaviors above								
Yes	447	78.2	76.2	71.4, 80.5	112	70.4	74.1	66.2, 80.6
No	125	21.8	23.8	19.5, 28.6	47	29.6	25.9	19.4, 33.8

**Table 2:** Socio-demographic characteristics, IPV, and sexual risk behavior among women who inject drugs in the Perempuan Bersuara study, Indonesia, unweighted estimates

Total (N = 731)	N	%
Socio-demographic and background variables		
Study city		
Greater Jakarta	572	78.2
West Java	159	21.8
Age (years)		
≤ 24	92	12.6
25–34	473	64.7
≥ 35	166	22.7
Education level (highest completed)		
Junior high school or lower	148	20.2
High school or higher	583	79.8
Employment status		
Unemployed	324	44.3
Employed (full-time, part-time, or contract work)	407	55.7
Housing status		
Unstable housing/homeless	39	5.3
Stable housing	692	94.7
Individual monthly income (in million Indonesian Rupiah, IDR)		
< 3.8 million IDR (approx. 285 USD)	398	54.5
≥ 3.8 million IDR (approx. 285 USD)	333	45.5
Primary source of monthly income (> 50%)		
Intimate partner	186	25.4
Family/relatives	127	17.4
Full-time employment	142	19.4
Casual/contract work	120	16.4
Trading sex	79	10.8
Other illicit/illegal activities (i.e., selling drugs, stealing)	77	10.5
Relationship status		
Currently married	283	38.7
Not currently married	448	61.3
Dependent children		
Yes	413	56.5
No	318	43.5
Type of drug injected (previous 12 months)		
Heroin	690	94.4
Buprenorphine	140	19.2
Pharmaceuticals^a^	29	4.0
Crystal methaphetamine	6	0.8
Type of drug non-injected (previous 12 months)		
Heroin	686	93.8
Crystal methamphetamine	491	67.2
Pharmaceuticals^a^	338	46.2
Cannabis	265	36.3
Ketamine	48	6.6
Self-reported HIV serostatus		
Reactive	341	46.7
Non-reactive	243	33.2
Unknown	147	20.1
Intimate partner violence (IPV) in the previous year		
Any type of IPV (psychological, physical/injurious and/or sexual)		
Yes	492	67.3
No	239	32.7
Psychological aggression		
Yes	435	59.5
No	296	40.5
Physical and/or injurious assault		
Yes	325	44.5
No	406	55.5
Sexual coercion		
Yes	217	29.7
No	514	70.3
Sexual risk behavior		
Unprotected vaginal or anal sex at last intercourse		
Yes	439	60.1
No	292	39.9
Sexually transmitted infection (STI) symptomatology (≥ 2)		
Yes	324	44.3
No	407	55.7
Number of sex partners (past 12 months)		
≤ 1	428	58.6
2–5	198	27.1
≥ 6	105	14.4
Endorsement of one or more of the sexual risk indices above		
Yes	559	76.5
No	172	23.5

^a^Pharmaceuticals include benzodiazepines (e.g., diazepam) and opiate-based medication (e.g., codeine, tramadol) used without a prescription

There were notable differences in the prevalence of IPV victimization across the two survey cities (Table 1). Bandung had a lower prevalence of any form of past-year IPV (55.9%; 95% CI 48.0, 63.5) relative to Greater Jakarta (68.9%; 95% CI 65.0, 72.6). Participants in Greater Jakarta reported higher levels of psychological aggression (58.8%; 95% CI 54.7, 62.8) as compared with those in Bandung (52.6%; 95% CI 44.7, 60.3). The prevalence of sexual coercion in Greater Jakarta (39.8%; 95% CI 35.7, 44.0) was nearly double that in Bandung (19.6%; 95% CI 13.7, 27.3). However, reported levels of physical and/or injurious assault were higher in Bandung (42.2%; 95% CI 34.8, 50.0) than in Greater Jakarta (38.8%; 95% CI 35.0, 42.8).

### Bivariate Associations Between IPV and HIV Sexual Risk Behavior

There were significant positive associations in bivariate analyses between the sexual risk behavior outcome and each form of IPV (Table 3): psychological aggression (OR 2.92, 95% CI 2.05, 4.16; p < 0.001), physical and/or injurious assault (OR 2.73, 95% CI 1.87,3.98; p < 0.001), and sexual coercion (OR 2.38, 95% CI 1.54,3.66; p < 0.001). Additionally, there were statistically significant associations at p < 0.1 between sexual risk behavior and several background variables: crystal meth use (OR 2.47, 95% CI 1.74,3.51; p < 0.001), HIV-positive status (OR 1.37, 95% CI 0.97, 1.94; p = 0.074), lower than high school educational attainment (OR 2.59, 95% CI 1.53, 4.39; p < 0.001), being currently married (OR 0.74, 95% CI 0.53, 1.05; p = 0.093), homelessness and/or unstable housing (OR 3.88, 95% CI 1.18,12.75; p = 0.026), age (OR 0.95, 95% CI 0.91, 0.98; p = 0.002), and survey city (OR 1.50; 95% CI 1.01, 2.23; p = 0.044). These variables were thus retained in subsequent multivariate models.

**Table 3:** Bivariate associations between IPV, sociodemographic and background variables, and sexual risk behavior among women who inject drugs in the Perempuan Bersuara study, Indonesia

Independent variables	Dependent variable: engaged in sexual risk behavior (*n = *559)		
	ORs	95% CIs	*p* value
Psychological aggression (ref. no)			
Yes	2.92	2.06, 4.16	≤ 0.001
Physical and/or injurious assault (ref. no)			
Yes	2.73	1.87, 3.98	≤ 0.001
Sexual coercion (ref. no)			
Yes	2.38	1.55, 3.66	≤ 0.001
Non-injection crystal methamphetamine use (ref. no)			
Yes	2.47	1.74, 3.51	≤ 0.001
Self-reported HIV status (ref. negative/unknown)			
Positive	1.37	0.97, 1.94	0.074
Survey city (ref. Bandung, West Java)			
Greater Jakarta	1.50	1.01, 2.23	0.044
Age (years)	0.95	0.91, 0.98	0.002
Education level (ref. high school and/or higher)			
Junior high school or lower	2.59	1.53, 4.39	≤ 0.001
Housing status (ref. stable housing)			
Unstable housing/homeless	3.88	1.18, 12.75	0.026
Employment status (ref. employed)			
Unemployed	0.89	0.63, 1.26	0.509
Individual monthly income (ref. ≥ 3.8 mill IDR)			
< 3.8 mill IDR (approx. 285 USD)	1.07	0.76, 1.52	0.680
Financial dependency (ref. no)			
Yes	1.03	0.70, 1.53	0.878
Relationship status (ref. not currently married)			
Currently married	0.74	0.53, 1.05	0.093
Dependent children or other dependents (ref. no)			
Yes	1.21	0.86, 1.70	0.278

*95% CI* 95% confidence intervals; *ORs* odds ratios

### Multivariate Associations Between IPV and HIV Sexual Risk Behavior

After adjusting for significant covariates, all three forms of IPV were associated with elevated odds of engaging in sexual risk behaviors (models 1–3, Table 4). Specifically, women who experienced psychological IPV were nearly three times more likely to engage in sexual risk behavior (OR 2.77; 95% CI 1.91, 4.03; p < 0.001) relative to women who did not experience such abuse. Furthermore, women exposed to physical and/or injurious IPV were at least twice more likely to engage in sexual risk behavior (OR 2.49; 95% CI 1.67, 3.70; p < 0.001) compared with women who were not exposed to physical and/or injurious IPV. Lastly, experiencing sexual IPV nearly tripled the odds of engaging in sexual risk behavior (OR 2.61; 95% CI 1.65, 4.13; p < 0.001). There were statistically significant positive associations between engaging in sexual risk behavior and the following covariates in all three multivariate models: crystal meth use, HIV-positive status, younger age, lower than high school educational attainment, and not being currently married.

**Table 4:** Multivariate associations between IPV, sociodemographic and background factors and HIV sexual risk behavior among women who inject drugs in the Perempuan Bersuara study, Indonesia

Independent variables	Dependent variable: engaged in sexual risk behavior (n = 559)		
	ORs	95% CIs	p-value
Model 1^a^			
Past-year psychological aggression (ref. no)			
Yes	2.77	1.91, 4.03	≤ 0.001
Non-injection crystal methamphetamine use (ref. no)			
Yes	2.26	1.54, 3.33	≤ 0.001
HIV serostatus (ref. non-reactive/unknown)			
Reactive	1.72	1.18, 2.53	0.005
Age (years)	0.96	0.92, 0.99	0.023
Education level (ref. high school and/or higher)			
Junior high school or lower	2.50	1.42, 4.41	0.002
Housing status (ref. stable housing)			
Unstable housing/homeless	1.80	0.53, 6.19	0.349
Relationship status (ref. not currently married)			
Currently married	0.54	0.36, 0.81	0.003
Survey city (ref. Bandung, West Java)			
Greater Jakarta	1.32	0.82, 2.12	0.249
Model 2^b^			
Past-year physical and/or injurious assault (ref. no)			
Yes	2.49	1.67, 3.70	≤ 0.001
Non-injection crystal methamphetamine use (ref. no)			
Yes	2.30	1.57, 3.37	≤ 0.001
HIV serostatus (ref. non-reactive/unknown)			
Reactive	1.65	1.13, 2.42	0.010
Age (years)	0.96	0.92, 0.99	0.022
Education level (ref. high school and/or higher)			
Junior high school or lower	2.46	1.40, 4.34	0.002
Housing status (ref. stable housing)			
Unstable housing/homeless	1.71	0.50, 5.86	0.392
Relationship status (ref. not currently married)			
Currently married	0.58	0.38, 0.86	0.007
Survey city (ref. Bandung, West Java)			
Greater Jakarta	1.39	0.87, 2.24	0.169
Model 3^c^			
Past-year sexual coercion (ref. no)			
Yes	2.61	1.65, 4.13	≤ 0.001
Non-injection crystal methamphetamine use (ref. no)			
Yes	2.43	1.66, 3.55	≤ 0.001
HIV serostatus (ref. non-reactive/unknown)			
Reactive	1.80	1.23, 2.63	0.002
Age (years)	0.96	0.92, 0.99	0.024
Education level (ref. high school and/or higher)			
Junior high school or lower	2.65	1.50, 4.67	0.001
Housing status (ref. stable housing)			
Unstable housing/homeless	2.88	0.85, 9.77	0.090
Relationship status (ref. not currently married)			
Currently married	0.56	0.38, 0.84	0.005
Survey city (ref. Bandung, West Java)			
Greater Jakarta	1.11	0.69, 1.77	0.664
Model 4^d^			
Past-year psychological aggression (ref. no)			
Yes	1.87	1.17, 2.99	0.009
Past-year physical and/or injurious assault (ref. no)			
Yes	1.53	0.93, 2.50	0.093
Past-year sexual coercion (ref. no)			
Yes	1.98	1.22, 3.21	0.006
Non-injection crystal methamphetamine use (ref. no)			
Yes	2.27	1.54, 3.35	≤ 0.001
Self-reported HIV status (ref. non-reactive/unknown)			
Reactive	1.70	1.15, 2.50	0.007
Age (years)	0.96	0.92, 1.00	0.040
Education level (ref. high school and/or higher)			
Junior high school or lower	2.53	1.43, 4.50	0.002
Housing status (ref. stable housing)			
Unstable housing/homeless	1.95	0.56, 6.78	0.293
Relationship status (ref. not currently married)			
Currently married	0.50	0.33, 0.76	0.001
Survey city (ref. Bandung, West Java)			
Greater Jakarta	1.23	0.76, 2.00	0.400

*95% CI* 95% confidence intervals, ORs odds ratios

^a^Model 1 includes past-year psychological aggression, controlling for past-year crystal methamphetamine use, self-reported HIV status, age, education level, relationship status, housing status, and survey city

^b^Model 2 includes physical and/or injurious assault, controlling for past-year crystal methamphetamine use, self-reported HIV status, age, education level, relationship status, housing status, and survey city

^c^Model 3 includes sexual coercion, controlling for past-year crystal methamphetamine use, self-reported HIV status, age, education level, relationship status, housing status, and survey city

^d^Model 4 includes all IPV dimensions (psychological, physical and/or injurious, sexual) in the same model, controlling for past-year crystal methamphetamine use, self-reported HIV status, age, education level, relationship status, housing status, and survey city. Cox-Snell R^2^ = 0.133; Nagelkerke R^2^ = 0.200

Model 4 (Table 4) included all dimensions of IPV and controlled for significant covariates. Psychological aggression (OR 1.87, 95% CI 1.17, 2.99; p = 0.009), and sexual IPV (OR 1.98; 95% CI 1.22, 3.21; p = 0.006) remained independently positively associated with sexual risk behavior, even after controlling for other IPV dimensions (i.e. physical and/or injurious). Furthermore, several background variables remained significantly positively associated with sexual risk behavior: crystal meth use (OR 2.27, 95% CI 1.54, 3.35; p < 0.001), HIV-positive status (OR 1.70, 95% CI 1.15, 2.50; p = 0.007), and lower than high school educational attainment (OR 2.53, 95% CI 1.43, 4.50; p = 0.002). Two covariates were negatively associated with engaging in sexual risk behavior: age (OR 0.96, 95% CI 0.92, 1.00; p = 0.040), and marital status (OR 0.50, 95% CI 0.33, 0.76; p = 0.001).

### Additive Effects

**Fig. 1:**
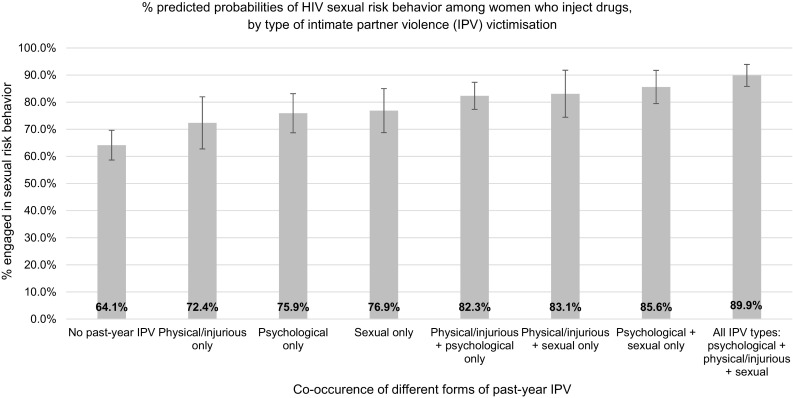
Marginal effects model testing for additive effects of different forms of intimate partner violence on sexual risk behaviour among women who inject drugs. Controls for self-reported HIV status, non-injection crystal methamphetamine use, age, relationship status, education level, housing status, and survey city

The predicted probabilities of the outcome when exposed to co-occurring forms of IPV victimization are displayed in Fig. 1. Strong additive effects were shown on women’s sexual risk behavior. The prevalence of sexual risk behavior was 64.1% among women who did not experience any form of past-year IPV. Among women exposed to any one of psychological, physical and/or injurious, or sexual IPV, 72.4–76.9% reported engaging in sexual risk behavior. Among women experiencing two co-occurring forms of IPV, levels of sexual risk behavior increased to 82.3–85.6%. With polyvictimization of all three forms of IPV, the percentage of women reporting sexual risk behavior escalated to 89.9%.

## Discussion

Findings from this study suggest that, when considered separately, psychological, physical, and sexual dimensions of IPV each has significant effects on sexual risk among women who inject drugs in Indonesia. This study found that at least 6 in 10 women who inject drugs were exposed to some form of past-year IPV, which is up to 24 times higher than IPV prevalence found in the general Indonesian female population [67]. Approximately three quarters of women across the two study cities engaged in HIV sexual risk behavior. It is especially concerning that nearly half of the women had STI symptoms, since the presence of STIs increases the infectiousness of HIV and facilitates HIV transmission [68, 69]. Furthermore, more than one third of women had multiple sexual partners, yet only 36.7% reported using a condom at last sex. These figures suggest a gendered vulnerability and risk: by comparison, among a national sample of mostly male injecting drug users in Indonesia, 51.6% reported using a condom at last sex [18]. Together, these findings highlight drug-using women’s considerable risk for contracting HIV and onwards transmission to their sexual and injecting partners, and perinatally to infants. For these urban, low-income women, IPV victimization could result in increased engagement in risky sexual behavior, and therefore in increased vulnerability to HIV. This risk was magnified by 26% when psychological, physical and/or injurious, and sexual forms of IPV co-occurred.

The finding that IPV is associated with sexual risk behavior is consistent with results from previous studies [8, 51, 70]. This association may be explained by women’s limited capacity to negotiate safer behaviours, particularly in abusive relationships. For instance, research with women in methadone treatment in the U.S. found that women who insisted that a partner use condoms were at elevated risk of IPV victimization, since partners tended to perceive such requests as a breach of gender role expectations, lack of trust, sign of infidelity or a threat to male dominance in the relationship. In the Indonesian context, fear of escalating partner aggression may lead some women to acquiesce to condomless sex and other risky behaviors, as has been substantiated by qualitative research with women who inject drugs in Central Java [25]. The link between experiencing IPV and elevated sexual risk-taking in Indonesia may be further shaped by social stigma, cultural norms and relationship power differentials [23]. Gendered cultural beliefs around placing care for an intimate partner above oneself, avoiding conflict, and preserving harmony in relationships may contribute to women’s inability to negotiate safer sex, even in situations when they feel they are at risk [23, 71]. Such gendered dynamics may also extend to the link between IPV and having multiple sexual partners. Previous studies have shown that the traumatic and economic consequences of being in an abusive relationship may steer women into other types of relationships, including survival sex work [8, 41, 46]. Women in intimate partnerships with drug-using partners may feel added pressure to trade sex and engage in risky encounters in order to maintain both their and their partners’ drug supply and to provide for their families [46, 72]. In the Indonesian context, this may include the practice of *turbo* (*tukar body)*, or trading sex with drug dealers in exchange for drugs. This practice is common among low-income, drug-using women and may be enforced by women’s partners as part of a gendered division of labour [19].

Crucially, psychological and sexual IPV victimization remained independently associated with HIV risk-taking behavior after controlling for other dimensions of IPV. This finding contributes to an emerging body of research showing that psychological aggression exerts comparable effects to physical and/or sexual forms of IPV [11]. A reduction in effect estimates was observed when all forms of IPV were included in the model, which could reflect any of the following: collinearity, confounding, or mediating relationships between the variables assessed. Given the small VIF scores detected for the model, overall collinearity is unlikely to have impacted the effect estimates. However, confounding through additional variables cannot be fully ruled out in cross sectional study analyses such as this one [63]. Two-way interactions were assessed between all model variables and none were found. We can speculate about potential mediating relationships in light of past research. Women’s crystal meth use may mediate the complex relationship between experiencing IPV and HIV risk, as using different types of drugs, including stimulants, has been associated with both violence victimization and unsafe sexual practices [51, 73, 74]. The association between IPV and HIV risk may be further mediated by women’s low socio-economic status, which could drive women to use drugs and trade sex in perilous environments where coercive sex is common and safe sexual practices are challenging to negotiate [75]. Furthermore, there is strong evidence for the role of mental health, particularly depression and post-traumatic stress disorder, in both perpetuating and mediating relationships between substance use, IPV, and HIV [76–78]. Mental health challenges can also play a role in increasing women’s substance use and in inhibiting their ability to discern and navigate risky situations [79]. Future research among women who use drugs in low- and middle-income settings has the opportunity to explore these and other potential mediating mechanisms which are associated with both IPV and HIV risk outcomes.

Moreover, our findings show that crystal meth use doubled the odds of engaging in sexual risk behavior. This finding confirms previous research from North America [55, 80] and contributes to a nascent body of evidence on the link between non-injection crystal meth use and elevated HIV transmission risk in Indonesia [81]. This finding is particularly alarming considering that Indonesia has seen a surge in crystal meth use in recent years [82], which may be contributing to an increase in overall HIV prevalence rates. Existing HIV prevention interventions for women who inject drugs in Indonesia should therefore be adapted to better meet the needs of poly-drug and crystal meth users.

Furthermore, HIV-positive status was associated with greater sexual risk-taking. Previous research with injecting populations has been inconclusive on the relationship between HIV sero-positivity and sexual risk, with studies finding both positive [83] and negative [84] associations. Our findings suggest that effective prevention of HIV among women who inject drugs and their intimate partners in Indonesia may benefit from including enhanced prevention efforts focusing on substance-using women who live with HIV.

In addition, younger women and women with a lower level of education were more likely to engage in sexual risk behavior. These findings support research from the U.S. suggesting that women’s educational and social disadvantage places them at higher risk of HIV transmission [5, 85]. Women’s education level plays an important role in perpetuating high-risk situations and gender power imbalances [85], which may be intensified in abusive and drug-involved relationships. Our finding that married women were less likely than their non-married counterparts to engage in sexual risk behavior is at odds with previous research in one regard. Specifically, studies have suggested that women in steady relationships, including those with high-risk partners, are more likely to engage in risky sex than those in casual or transactional partnerships [8].

The findings in this paper suggest that IPV could also influence other HIV-related health outcomes and merits further investigation in Asia and other regions with injection-driven epidemics. For instance, the same mechanisms that heighten the risk of contracting sexually-transmitted HIV appear to also elevate women’s injecting risk (i.e. syringe sharing and borrowing) and should be considered in future analyses [86, 87]. Furthermore, emerging research among drug-using women in Malaysia points to the adverse effects of IPV, co-occurring mental health challenges, and low social support on HIV testing and/or monitoring outcomes [53]. In the context of accumulating research on the detrimental effect of overlapping syndemics in perpetuating health-related disparities among women who use drugs [5], future research with drug-using women in Indonesia may benefit from investigating associations and mechanisms linking IPV with a broader range of HIV risk and treatment outcomes such as viral load, enrolment, and adherence to antiretroviral treatment.

The results of this study should be considered in the context of several limitations. First, the cross-sectional design prohibits assessing causality, highlighting the need for follow-up research employing longitudinal designs. Second, the use of self-report may be subject to recall and reporting bias, including the tendency to under-report stigmatised behaviors [88]. Longer recall periods (e.g. lifetime, past year) carry a higher risk of erroneous reporting than measures requiring shorter recall periods (e.g. past week, month, or three months). Thus, the findings are vulnerable to potential biases related to recall bias around sexual and injecting behaviors, which were assessed for the preceding 12 months. This limitation was minimised through the use of a peer recruitment strategy [89] shown to enhance the validity and reliability of data by improving rapport and trust and enabling participants to provide more honest responses [90]. Third, findings should be carefully interpreted in light of potential biases associated with RDS analytical methods. Since RDS depends on social networks for referral, some sub-populations of drug-using women (i.e. women injecting only with one partner) might be underrepresented in this sample due to having limited networks within the broader population. Furthermore, because RDS recruitment starts with purposively selected seeds that may or may not accurately represent the underlying network structure of the population, there is a risk that the resulting sample may be more representative of the characteristics of the seeds rather than the those of the target population, resulting in a form of selection bias [91]. We attempted to minimize the potential impact of seed selection on the estimates by monitoring convergence plots for key variables throughout data collection to see whether the estimates appeared to stabilize with successive waves of recruitment. Given that recruitment chains at both study sites converged to a sampling equilibrium well in advance of attaining the full sample size, we concluded that bias associated with the non-random recruitment of seeds was substantially eliminated. We paid particular attention to monitoring recruitment chains in Bandung, where only two seeds generated 100% of the sample size in that city and nearly 22% of the cumulative sample across the two study sites. The recruitment chains were long enough to ensure that there was no correlation of characteristics to the seed with the outcome of recruitment, and the recruitment process penetrated deeply into the population of women who inject drugs in Bandung. In addition, there was no evidence of differential recruitment activity as observed by low homophily scores and minimal differences in mean personal degrees for all variables of interest, indicating that lingering bias associated with seed dependence was unlikely to affect the estimates. Finally, a key theoretical assumption in RDS is that each sample comprises a single network component [60]. Although RDS-adjusted estimates were reported separately for each city, multivariate analyses were performed on the combined, unweighted dataset in order to retain the power and precision corresponding to the original calculated sample size. Therefore, while the city-level prevalence estimates fulfilled RDS theoretical assumptions, findings from the regression and marginal effects analyses may not be generalizable to other settings.

Notwithstanding these limitations, the data presented here constitute the best currently available evidence on correlates of HIV sexual risk behavior in a diverse sample of Indonesian women who inject drugs. This study has important programmatic implications. Our findings provide new insights into the sexual risk associations of different forms of IPV among women who inject drugs in Asia. Longitudinal data are needed to test the temporal relationships of associations observed in this study, and assess potential causal mechanisms to be subsequently addressed through intervention research. Such analyses would be the next step to informing evidence-based interventions and service provision. As a first step, IPV prevention and screening targeting women who use drugs in Indonesia should be integrated within existing HIV prevention and harm reduction programs. Possible settings for service integration include methadone maintenance clinics, community health centers providing HIV prevention services, and community-based drug treatment facilities. Ultimately, this study’s findings demonstrate an urgent need for the optimization of HIV prevention interventions to better respond to the high prevalence of IPV and HIV risk among Indonesian women who inject drugs.

## Electronic supplementary material

Below is the link to the electronic supplementary material.

Supplementary Appendices (DOCX 1354 kb)
