# Supplementary material for: Intimate Partner Violence and HIV Sexual Risk Behaviour Among Women Who Inject Drugs in Indonesia: A Respondent-Driven Sampling Study
Source: AIDS Behav. 2018 Jun 11;22(10):3307–23. doi: 10.1007/s10461-018-2186-2 (PMC6154010; doi:10.1007/s10461-018-2186-2)
Supplement: Supplementary file 1 — Supplementary Appendices (DOCX 1354 kb) [file 10461_2018_2186_MOESM1_ESM.docx]

**Supplementary Appendix I:** Convergence plots depicting the weighted cumulative prevalence of select outcome and analysis variables across participants at each wave of recruitment. Prevalence (%) is shown on the Y-axis and recruitment waves on the X-axis. The horizontal dashed line indicates the estimated weighted proportion based on the complete sample.

**Fig 1. Greater Jakarta metropolitan area, Indonesia (N=572)**


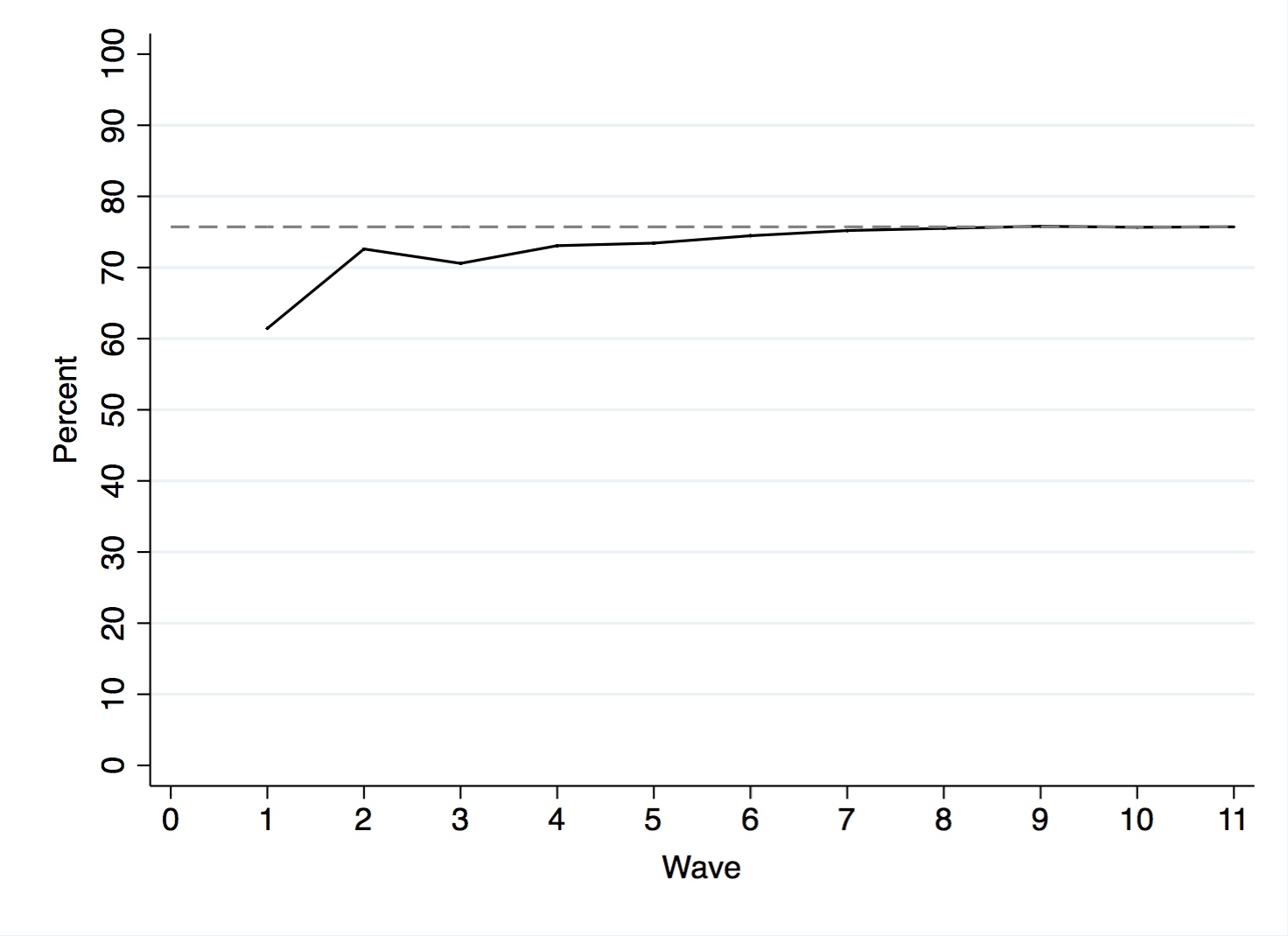

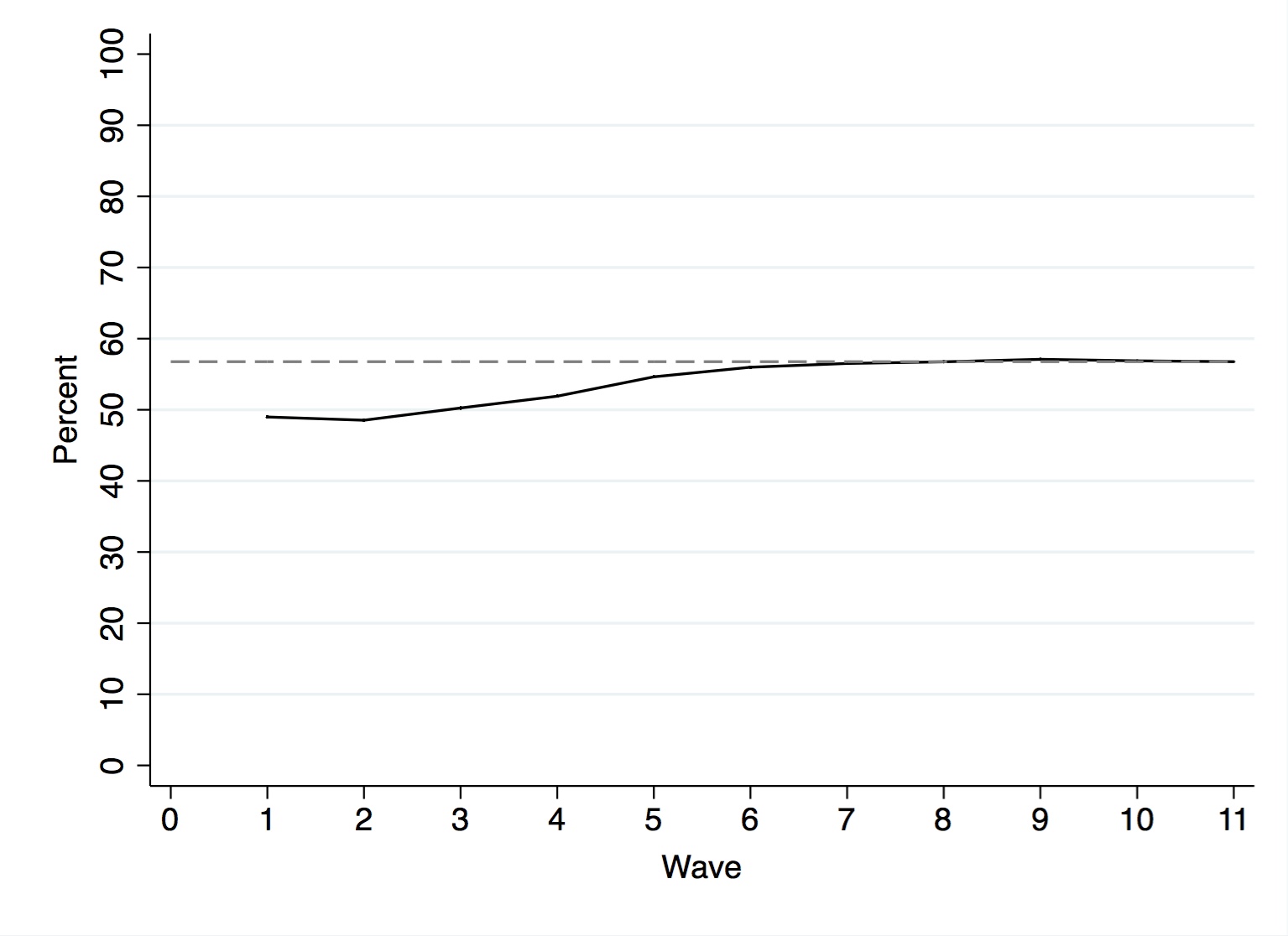
(a) Sexual risk behavior. (b) Past-year psychological aggression.


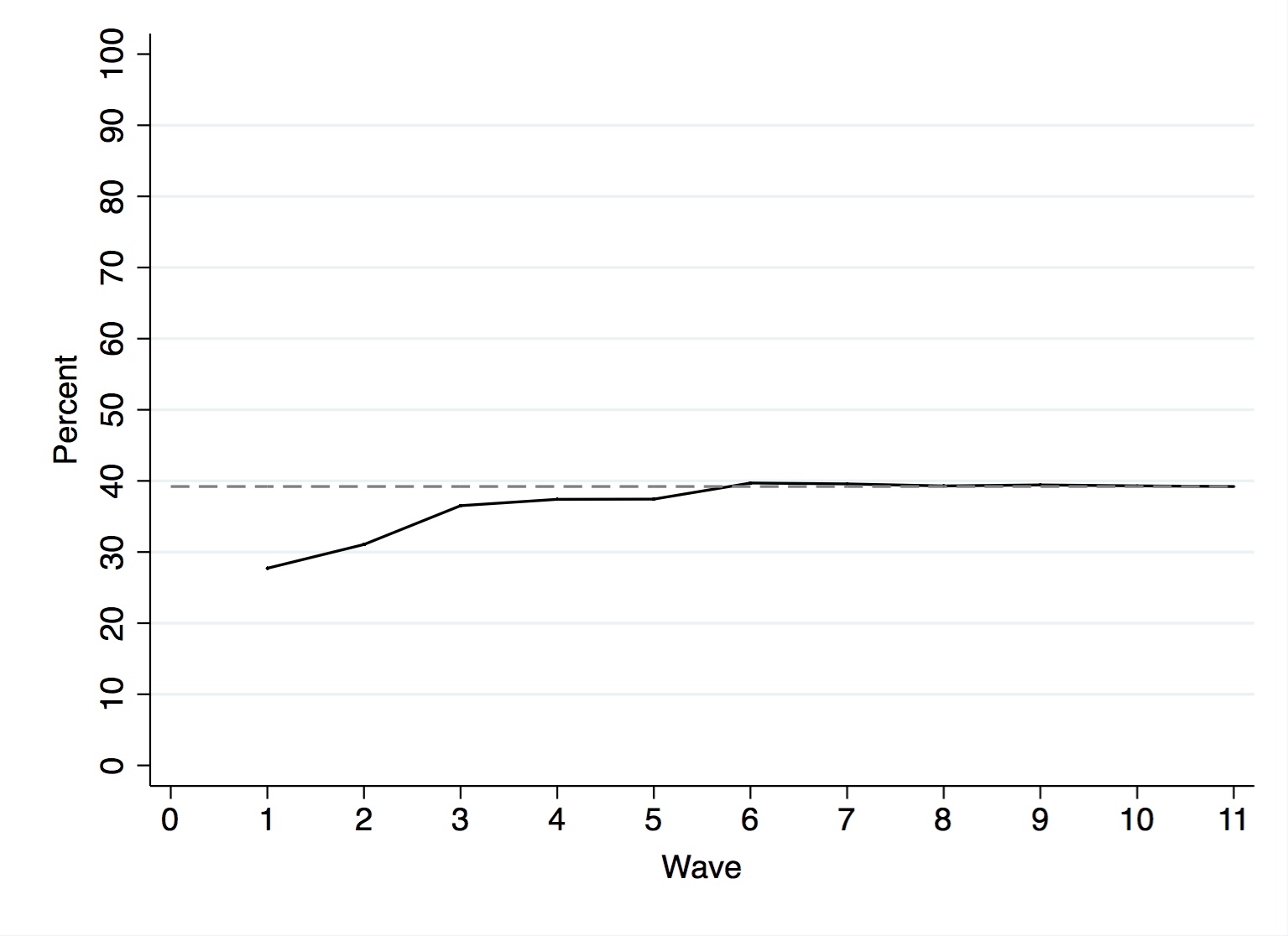
(c) Past-year physical and/or injurious assault. (d) Past-year sexual coercion.


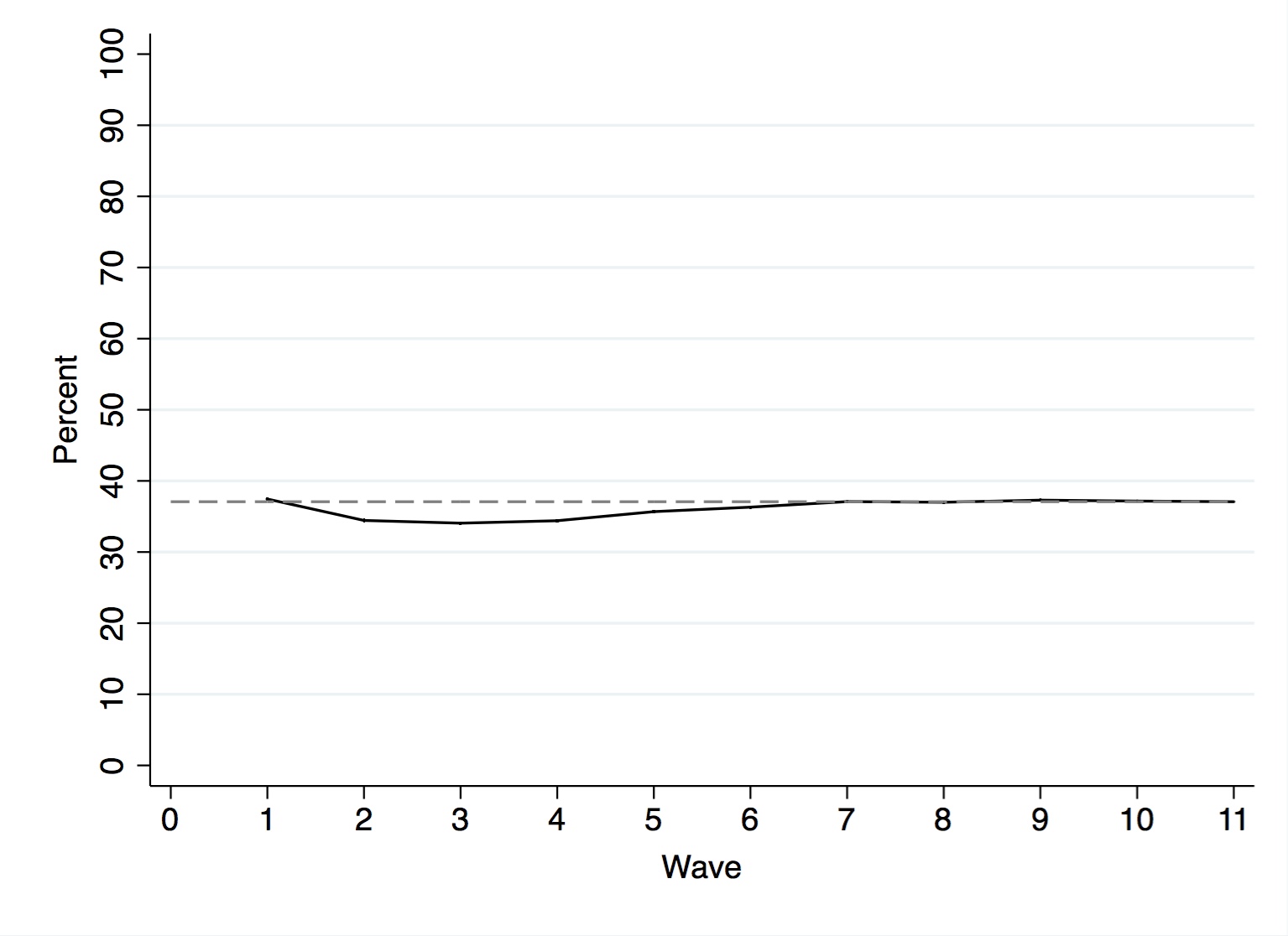


**Fig 2. Bandung, West Java, Indonesia (N=159)**


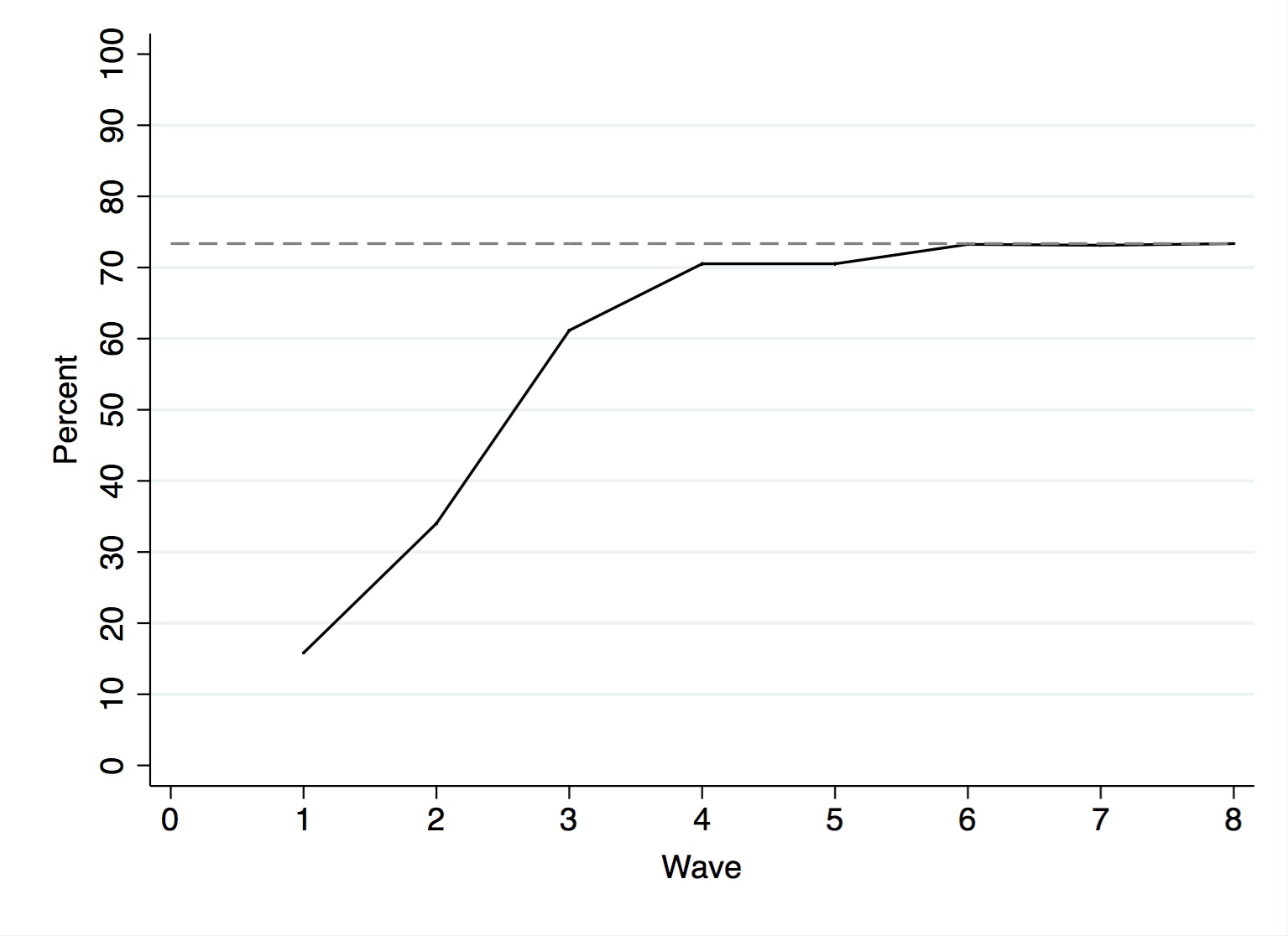

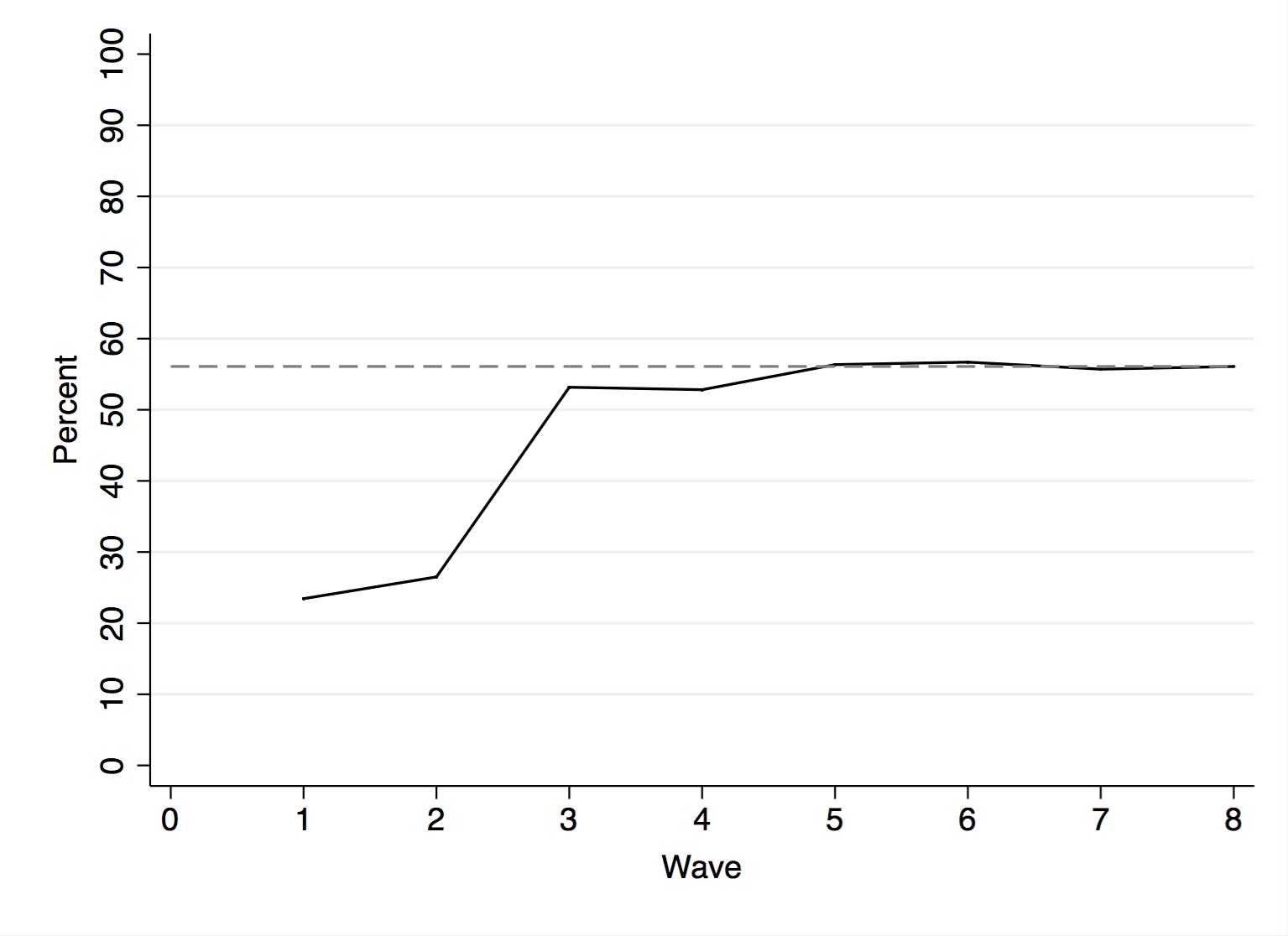
(a) Sexual risk behavior. (b) Past-year psychological aggression.


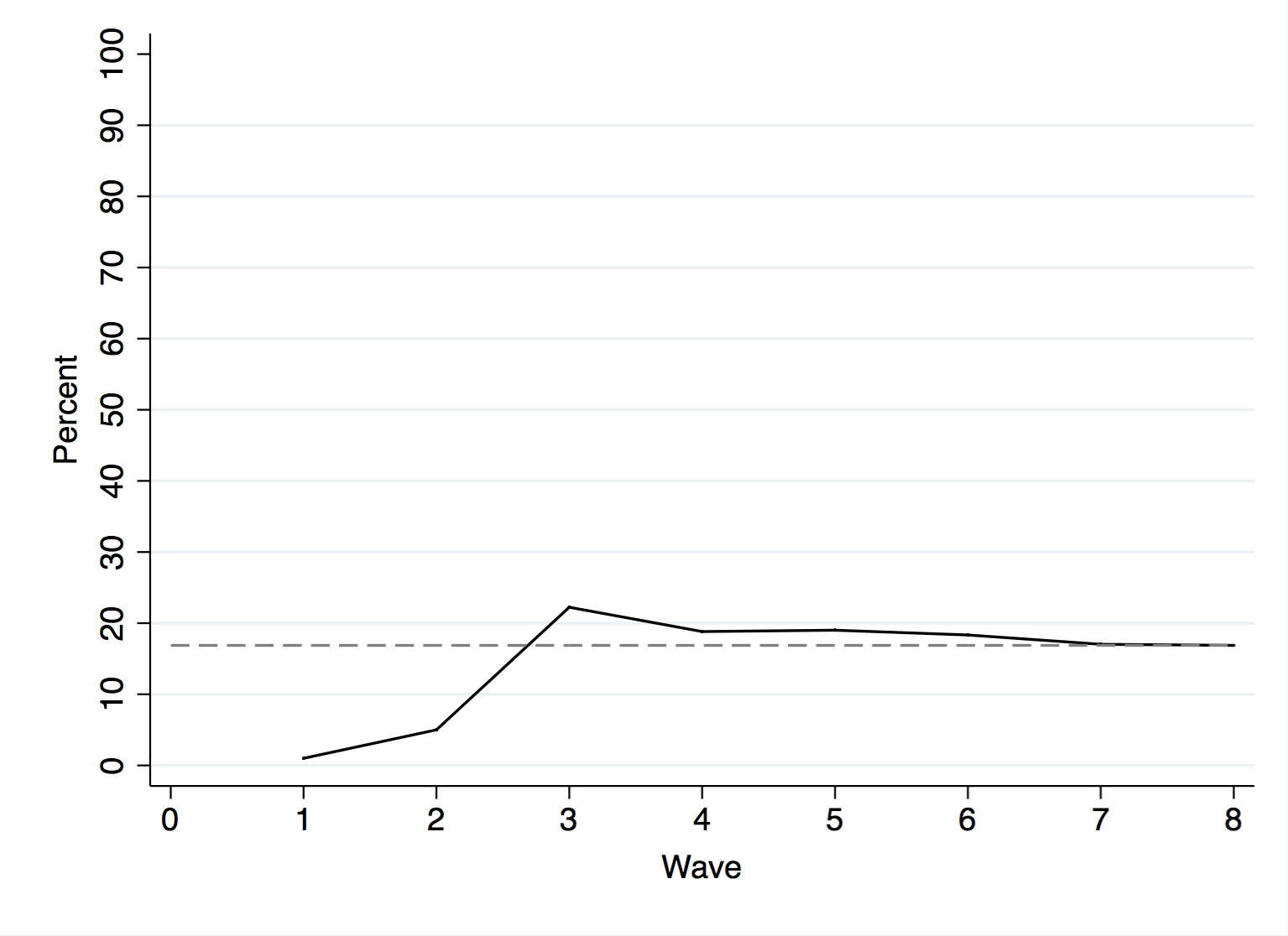
(c) Past-year physical and/or injurious assault. (d) Past-year sexual coercion.


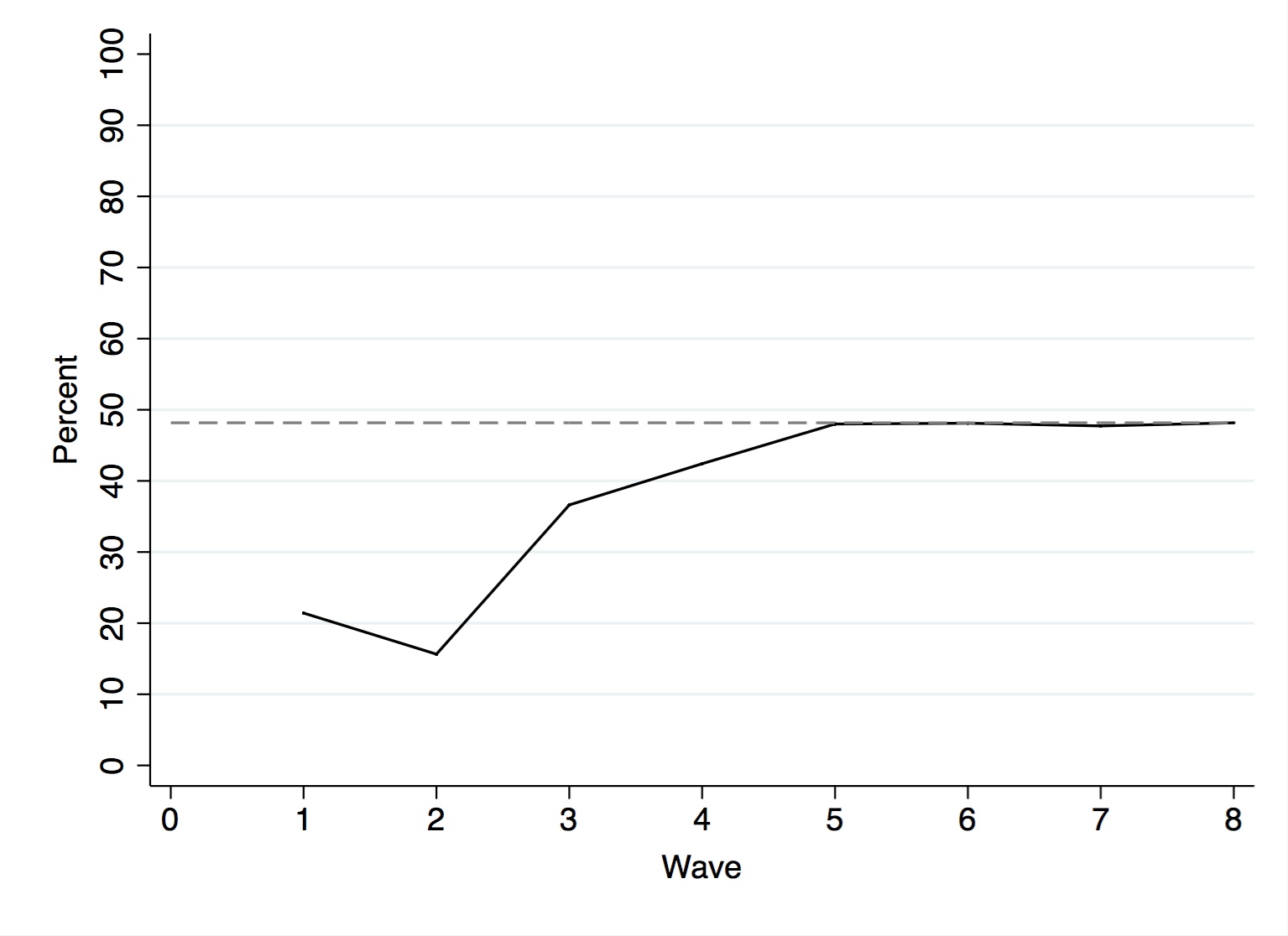


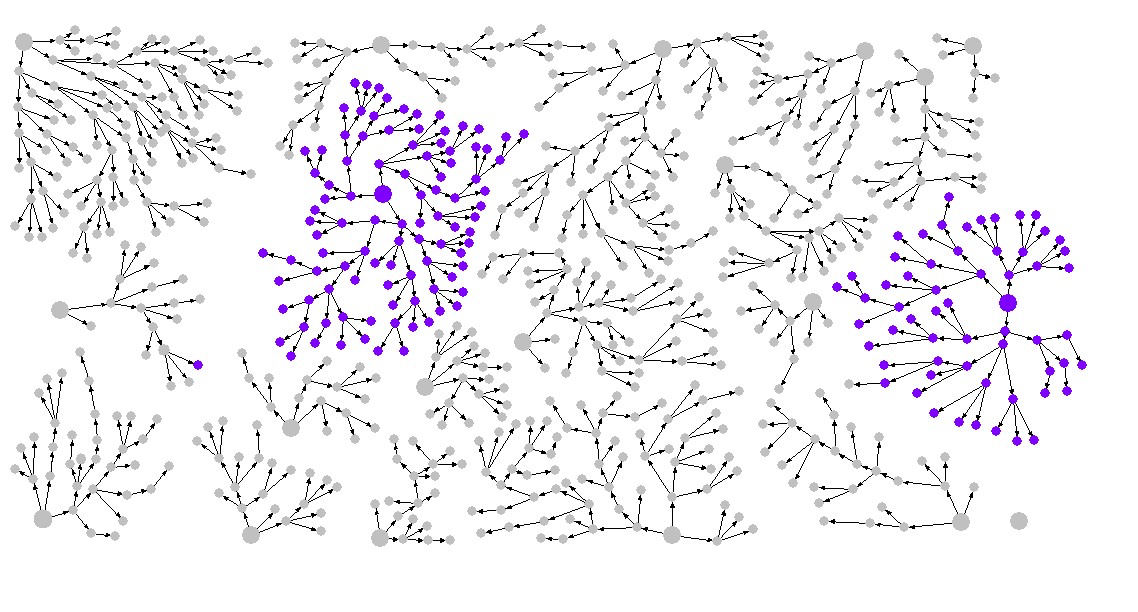
**Supplementary Appendix II**: Network structure for sample of 731 women who inject drugs recruited by the Perempuan Bersuara study, Indonesia. Larger circles represent seeds (initial participants, or wave 0); smaller circles represent peers recruited in successive waves. Purple represents participants recruited from Bandung, West Java; grey represents participants from Greater Jakarta. A bottleneck is observed across the two survey sites, such that the majority of participants were clustered in either Greater Jakarta or Bandung, forming two isolated geographical components with minimal across-group recruitment.
